# Discrete Element Simulation Study of the Accumulation Characteristics for Rice Seeds with Different Moisture Content

**DOI:** 10.3390/foods11030295

**Published:** 2022-01-22

**Authors:** Jinwu Wang, Changsu Xu, Xin Qi, Wenqi Zhou, Han Tang

**Affiliations:** 1 College of Engineering, Northeast Agricultural University, Harbin 150030, China; jinwuw@neau.edu.cn (J.W.); ChangsuXu@neau.edu.cn (C.X.); XinQi@neau.edu.cn (X.Q.); zwq@neau.edu.cn (W.Z.); 2 Key Laboratory of Crop Harvesting Equipment Technology of Zhejiang Province, Jinhua Polytechnic, Jinhua 321007, China

**Keywords:** natural repose angle, point source, velocity characteristics, mechanical characteristics, distribution

## Abstract

To study the accumulation characteristics of rice seeds with different moisture content, an accurate model of rice seeds was established by 3D scanning technology. The accumulation state of rice seeds by the “point source” accumulation method was analyzed by proportioning and measuring the simulation parameters with different moisture content. The accumulation process was simulated at 10.23%, 14.09%, 17.85%, 21.77%, 26.41% and 29.22% moisture content, respectively. The velocity and force state of the seeds were visually analyzed by using the accumulation process with a moisture content of 29.22%. The accumulation process was divided into four stages according to the velocity characteristics of the seeds. The average force and kinetic energy of the rice seeds outside the cylinder were obtained, and the average force of the rice seeds outside the cylinder was proved to be the direct cause of the velocity change during the accumulation process. The mechanical characteristics of rice seeds in the quasi-static accumulation stage were partitioned and systematically analyzed. The force distribution of the “central depression” structure of rice seeds with a moisture content of 10.23%, 14.09% and 17.85% on the horizontal surface appeared. The higher the moisture content of rice seeds, the more likely the typical “circular” force structure appeared, and the more uniformly the force on the horizontal surface was distributed in the circumference direction.

## 1. Introduction

The accumulation is one of the most common states of existence during the production, storage and processing of rice seeds. It is a highly dynamic and variable system with no viscous connection between seeds [1]. As a complex nonlinear system, the seed medium responds to various perturbations by “self-organizing” with changes in external factors, and its unevenly distributed internal chain-like mechanical structure allows it to exhibit many singularities like both solid and liquid [2]. Agricultural engineering and food engineering are the most widely used fields of grain systems. Grain accumulation is involved in the storage of materials, subsequent processing and the development of related equipment. The research on the accumulation characteristics of grain systems has important basic scientific significance and engineering application value [3].

Due to the dual behavior of solid and liquid, there are interactions between grain and granary before accumulation, such as blockage, arching, funnel flow and unstable flow [4,5]. The interaction between grain and granary affects the occurrence of accumulation, and the blockage problem has been widely concerned by many scholars. Wan et al. [6] studied the influence of orifice shape on particle velocity through the combination of simulation and experiment. Zaki et al. [7] explored the effects of different hopper shapes on grain discharge speed and efficiency, and established the relationship between particle speed and flow at the outlet of the silo. The lower the particle velocity and flow rate at the outlet of the silo, the easier the blockage problem is. Liu et al. [8] analyzed the relationship between silos with different orifice sizes and arch structure, and established the orifice size prediction model of curved fabric buckets. Xiao et al. [9] explored that the arch structure of particle flow at the outlet is the main cause of blockage from the perspective of particle dynamics. Ahmadi et al. [10] studied that the friction coefficient of particles has an important influence on the size formation of arch structure. The above research focuses on the interaction between grain particles and granaries, but there is little research on the dynamic characteristics of the grain itself in the process of unloading. The dynamic and mechanical characteristics of particles in the process of stacking are of great significance to guide the safe storage of grain. After the formation of the accumulation, the particle system will show a certain static and stable state, in which the “central depression” phenomenon is one of the key elements of the study. The maximum normal force at the bottom of the accumulation does not always occur at the center of the accumulation but may occur at the periphery of the center of the accumulation [11,12]. Horabik et al. [13] studied that the pressure of particles with different shapes on the bottom presented different distribution states. The phenomenon of “central depression” was directly manifested by significant differences in the distribution of contact forces. The contact force between particles was strongly correlated with the frictional characteristics of the surface of the particles [14]. Shi et al. [15] explored the effects of particle sliding friction and rolling friction on the accumulation process. In addition, Cao et al. [16] showed that the tangential force was higher when the bottom surface roughness was larger, and the corresponding “central depression” of the normal force was more obvious. Horabik et al. [17] studied the effects of stress distribution and contact friction on the “central depression” phenomenon. Carlevaro et al. [18] studied the formation of force chains and the arching mechanism in the accumulation of the particles and showed that the structure of the arching force chain supported the seed accumulation and carried most of the acting forces in the accumulation.

The non-transparency of the particle system led to the fact that the mechanical characteristics of the accumulation cannot be directly observed through tests. Meanwhile, the large amount of the particle material in engineering cannot be directly analyzed and determined, which largely limited the development of bulk mechanics. With the progressive development of computer simulation technology and similarity theory, the discrete element method has become an effective numerical simulation method to study the behavior of particle systems [19]. In the field of agricultural engineering and food engineering, the discrete element method is widely used in solving the movement characteristics of materials in machines, such as the screening status of grain particles in combine harvesters [20], gas and solid coupling simulation test [21], and the dynamic changes of grain in the rice milling machine [22]. In the discrete element numerical simulation, accurately obtaining the physical parameters of materials is the key to effectively exploring the particle motion and mechanical properties. The physical parameters mainly include material properties and interaction properties [23]. The different moisture content will lead to the change of physical properties of materials, which directly affects the accuracy of simulation results [24].

The spherical seeds with a single physical characteristic were mostly used by the discrete element simulation process. The physical characteristic of grain crops was changed significantly as their surface irregularities and different moisture content, which can cause variability in the mechanical characteristic during the accumulation process [25]. In this paper, the simulation model was accurately established based on rice grain by 3D scanning technology, and the numerical values of simulation parameters were explored through experiments with different moisture content. The accumulation state and mechanical properties of rice grains under different moisture content were studied by the discrete element method.

## 2. Materials and Methods

### 2.1. Parameter Determination

The characteristics reflected by rice with different moisture content are mainly manifested in the differences of physical parameters such as the density, modulus of elasticity, Poisson’s ratio, etc., [26]. Taking the rice grains of “Longjing 29” widely planted in the main rice producing areas of China as the research object, the 1000 grains were selected to measure and analyze their triaxial dimensions by using vernier calipers (Ningbo Deli tools Co., Ltd., Ningbo, China). The grain length is mainly distributed between 5–6 mm, the width is mainly distributed between 1–2 mm, and the thickness is mainly distributed between 1–2 mm. The average values of the triaxial dimensions are 5.63 mm, 2.05 mm and 1.80 mm, respectively.

The moisture content of rice seeds during the harvesting period was concentrated in the range of 10.23–29.22%. To achieve the test range of moisture content for the ideal rice seeds, the undamaged rice seeds of 1000 were artificially calibrated by the water supply method [27]. Each group of parameters was repeated three times, and the results were averaged. The main parameters of rice grain include moisture content [28], density [29], elastic modulus [30], static friction coefficient and dynamic friction coefficient [31], collision recovery coefficient [32] and Poisson’s ratio [33]. The parameter measuring methods are reported in Supplementary Materials (Table S1).

The rice seeds always interact with mechanical parts during production, storage and processing. Therefore, the mechanical contact material was set to be Q235 steel with the following physical characteristics: the density of 7850 kg/m^3^, the modulus of elasticity of 212 MPa and the Poisson’s ratio of 0.31. The results of the physical parameters of the rice seeds with different moisture content and the contact mechanical parameters with Q235 steel are shown in Table 1.

The effects of different moisture content on physical characteristics were evaluated by SPSS statistics 22.0 (SPSS, Chicago, IL, USA). Analysis of variance (ANOVA) was performed with Duncan’s multiple comparison to assess the significance level with a threshold of *p* < 0.05. The results were expressed as the mean ± standard error. Each replicate (*n* = 3) was taken as a random effect in this model.

### 2.2. Discrete Element Numerical Simulation Scheme

The overall structure of the rice seeds is complex. To accurately establish the model of the rice seeds, the rice seeds with approximate average values of the three axes dimensions were selected (5.58 mm, 2.05 mm and 1.83 mm). The Reeyee X5 3D laser scanner (Nanjing Weibu 3D Technology Co., Ltd., Nanjing, China) was used to extract the 3D geometric feature parameters of the rice seeds. A 3D laser scanner projected gratings onto the surface of the rice grain. According to the shape of the fringes changing with the curvature, the spatial coordinates of each point of the surface were accurately calculated by using the orientation method and the triangulation method. The 3D point cloud data were generated. Shading, denoising, point cloud registration, point cloud triangulation, merging and model correction were carried out in turn. Using automated reverse engineering software to convert scan data into an accurate digital model, the ideal 3D scanning model of rice grain was obtained. This 3D laser-scanned rice grain model was imported into EDEM and filled with spherical element particle polymerization [34]. The process of discrete element modeling of rice seeds is reported in Supplementary Materials (Figure S1).

The surface of rice seeds is smooth without adhesion. To accurately represent the contact characteristics of rice seeds, the Hertz–Mindlin (no-slip) model was used to construct the contact between rice seeds and rice seeds and between rice seeds and steel plates [35].

Place the cylinder vertically on the horizontal plane (the cylinder and the horizontal plane are made of Q235 steel). The diameter of the cylinder is 50 mm and the height is 150 mm. The *Z*-axis direction was selected as the direction of gravity with reference to the constructed model coordinates, and its value was set to −9.81 m/s^2^. The “falling rain method” was used to make the rice seeds accumulate naturally in the cylinder with zero initial velocity. The total number of rice seeds was set to 4000. When the rice seeds were completely settled in the cylinder, it started to lift the cylinder at a speed of 0.05 m/s. Until the rice seeds in the cylinder were naturally scattered into an accumulation under the action of gravity, the simulation was stopped after the rice seeds state of the accumulation was completely stationary and stable. The Rayleigh time step was determined jointly by the software based on parameters such as the seed radius and the density [36]. The Rayleigh time step was finally determined to be 20% and the total simulation time for each group was set to 3 s. The simulations of six groups for rice seeds were conducted with different moisture content, and this method was used for each group in this paper.

### 2.3. Comparative Verification of Accumulate Test

To verify the accuracy of the established discrete element model of rice seeds before simulation, the natural rest angle was measured by comparison between EDEM simulation and bench test. For each group, the 4000 rice seeds of “Long Japonica 29” with different moisture content were selected as the test varieties. The cylinder and horizontal surface of the same size and material were selected, and the same speed was used to lift the cylinder to ensure the consistency between EDEM simulation and bench test.

To accurately determine the natural rest angle of rice seeds, the accumulation of rice seeds in three-dimensional space was photographed, and the MATLAB software was used to process the image noise, grayscale and binarization [37].

## 3. Results and Discussion

### 3.1. Results of Comparative Verification for Accumulate Test

The unilateral contour curve of rice seed accumulation was extracted and the ratio of the vertical to horizontal pixel values of the curve was used to determine the magnitude of the natural rest angle. The results of the bench test and simulation test of natural rest angle for rice seeds with different moisture content are shown in Supplementary Materials (Figure S2).

**Figure 1:**
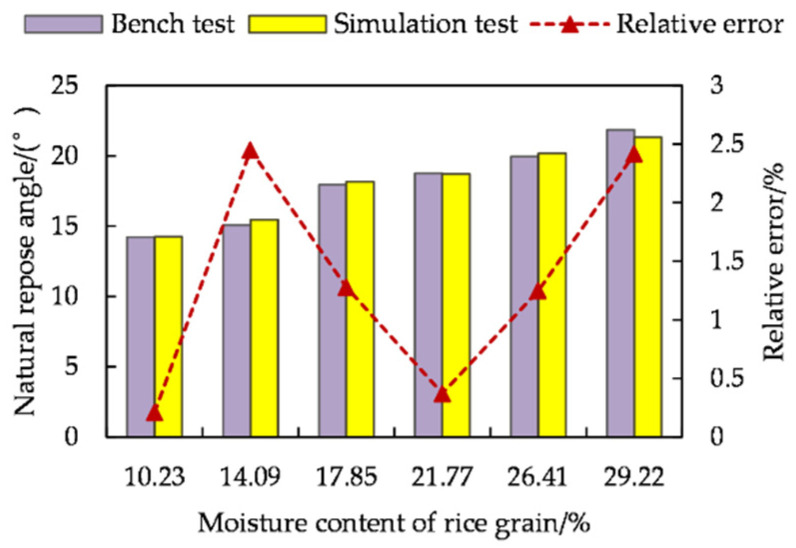
Natural rest angle of rice seeds with different moisture content.

The natural rest angle of rice seeds with different moisture content was analysed (Figure 1). The natural rest angle increased gradually with the increase of moisture content. This was mainly due to the fact that the higher the moisture content, the higher the friction between seeds, resulting in poor mobility of the population. The overall results of the comparison between the natural rest angle bench test and the simulation test for rice seeds with different moisture content had certain errors, and the maximum error was 2.45%.

This was mainly due to the fact that the rice seed metamodel consists of multiple small spheres aggregated to increase the surface area of rice seeds, and the frictional force at each contact part of adjacent seeds increased accordingly in the free accumulation process. Meanwhile, the overall shape of rice seeds in the bench test showed some variability. Within the allowable range of error, it showed that the discrete element model of the rice seed was accurate and effective, which laid a foundation for the study of mechanical characteristics of rice seed in the later stage of the accumulation.

### 3.2. Velocity Distribution in the Accumulation Process of Rice Seeds

**Figure 2:**
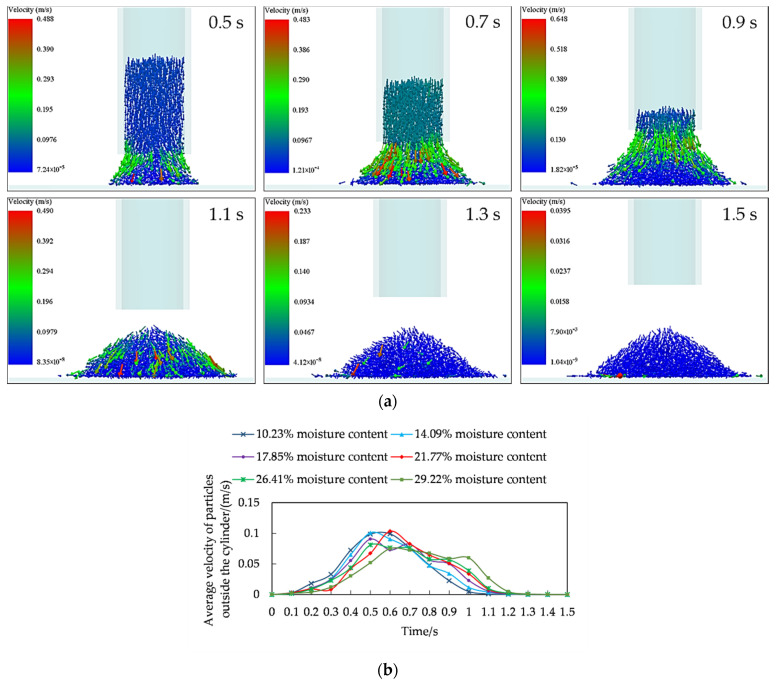
Characteristics of rice seeds accumulation velocity with the moisture content of 29.22%: (**a**) velocity distribution in the accumulation process; (**b**) average velocity of the rice seeds outside the cylinder at different moments.

To explore the velocity distribution of rice seeds in the accumulation process, the accumulation process of rice seeds with 29.22% moisture content as an example was taken. The velocity distribution in the accumulation process was deeply analyzed (Figure 2a).

The seeds gathered at the mouth of the cylinder and formed a column of material flow out from the bottom at the initial stage of accumulation for rice seeds (0–0.5 s). When the seeds reached the horizontal surface of the substrate, the seeds gradually accumulated in a cone shape at the center of the substrate. The speed of the inner layer of seeds was small to form the inner static support structure, and the outer layer of seeds had a larger speed and gradually stayed on the surface of the cone to expand outward at this time. This stage was called the substrate support forming stage. With the increase of time (0.5–0.9 s), the basal accumulation area of rice seeds kept expanding, and the number of outer seeds with higher velocity increased. This was mainly due to the continuous accumulation of the seeds layer, and the continuous seeds flow still fell heavily under the action of the gravity in the internal cylinder, which had a very strong disturbance effect on the seeds that were about to accumulate at rest. The seeds were forced to continue to roll with the flow of seeds. This stage was called the expansion accumulation stage, which was characterized by large seeds speed as a whole and rapid accumulation shape. The rice seeds had completely fallen out of the cylinder at the moment of 0.9–1.3 s, and the rice seeds in the accumulation process had no constraints and limitations of the cylinder. The rice seeds were freely scattered and shaped under the action of gravity, at which time the speed of the rice seeds gradually decreased. This stage was called the free accumulation stage. The accumulation process of the rice seeds was completed and the morphology of the accumulation no longer changes after 1.3 s. The accumulation of the rice seeds at this stage had completely stabilized. This stage was called the quasi-static accumulation stage.

To continuously quantify the velocity distribution of rice seeds with different moisture content during the accumulation process, the average velocity of the seeds outside the cylinder (the seeds formed the accumulation) at different moments was compared and analyzed with the accumulation time as the horizontal coordinate and the average velocity of the seeds as the vertical coordinate (Figure 2b). The average velocity of rice seeds with different moisture content showed a trend of increasing first and then decreasing during the accumulation process. When the moisture content was 10.23%, the average speed of rice seeds first rose to the maximum value. With the increase of moisture content, the average speed rosed to the maximum value in turn. The higher the moisture content, the later the average speed rosed to the maximum value. When the average speed of rice seeds reached the maximum value, the average speed of rice seeds with a moisture content of 10.23% firstly decreased and quickly reduced to 0. With the increase of moisture content, the average speed decreased to 0 in turn. The higher the moisture content, the later the time of the average speed decreased to the lowest value shifted. This may be due to the fact that as the moisture content increased, the friction on the surface of the rice seeds increased. Both the friction with the inner wall of the cylinder and the friction with the seeds produced a certain hysteresis effect, resulting in a longer time between the base-supported forming stage and the expanded accumulation stage in the accumulation process, and a corresponding lag between the free accumulation stage and the quasi-static accumulation stage.

### 3.3. Analysis of the Mechanical Characteristics of Rice Seeds during Accumulation

**Figure 3:**
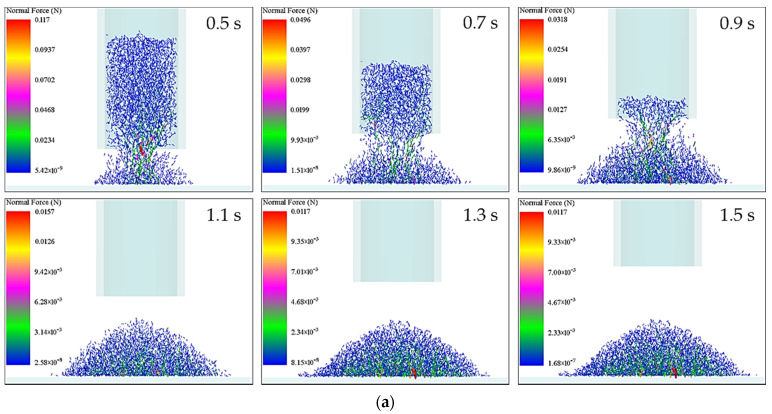

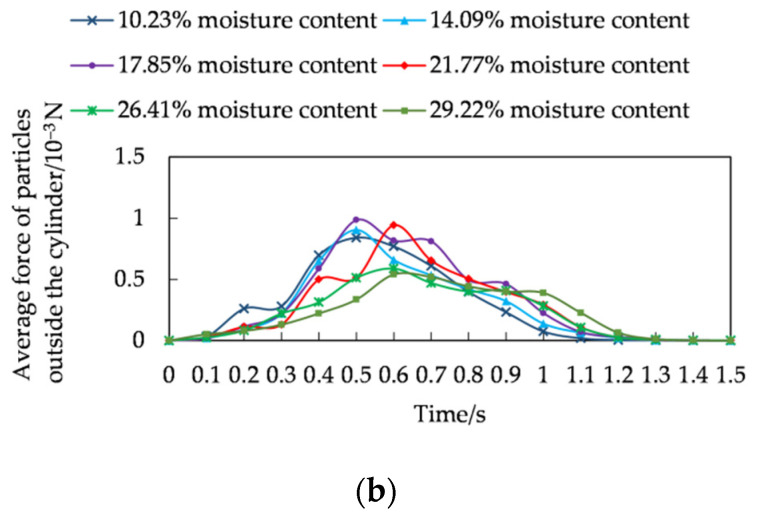
Force of rice seeds accumulation with 29.22% moisture content: (**a**) force distribution; (**b**) average force of the rice seeds outside the cylinder at different moments.

To further analyze the force situation during the accumulation of rice seeds at different moments, the accumulation process of rice seeds with a moisture content of 29.22% was taken as an example, and the force state of the inner and outer layers of the accumulation of rice seeds was visually analyzed by longitudinal section along the diameter direction of the accumulation of rice seeds. The force distribution in the accumulation process was deeply analyzed (Figure 3a).

In the substrate support forming stage, the inter-seed force was small and relatively uniform in the outer layer of the accumulation, while the local force between the seeds was larger in the inner part of the accumulation. A few seeds were supporting the weight of the whole accumulation and gradually forming the accumulation form.

When the rice seeds entered the expansion accumulation stage, the maximum force between the seeds gradually decreased with time and still occurred inside the accumulation. This was mainly due to the fact that the inside seeds were not only subjected to their own gravity, but also to the pressure of the seeds falling from the inside cylinder.

When the rice seeds entered the free accumulation stage, the maximum force between the seeds continued to decrease, but the maximum force occurred in the area of the inner bottom of the seed accumulation in contact with the horizontal surface. When the rice seeds entered the quasi-static accumulation stage, the maximum force between the seeds no longer changed, and the maximum force area still occurred at the bottom of the inner layer where the seeds were in contact with the horizontal surface. The force gradually decreased in a radial pattern from the inner layer to the outer layer of the seeds. The force in the outer layer was the smallest, which was mainly due to the fact that the outer layer of seeds was free only by its own gravity and the support force of the seed accumulation. At this time, the overall force on the accumulation reached equilibrium, and the accumulation state no longer changes.

To quantitatively analyze the distribution of accumulation forces on rice seeds with different moisture content during the accumulation process, and also to study the influencing factors on the variation of accumulation speed of rice seeds with different moisture content, the forces on seeds outside the cylinder (seeds forming the accumulation) were compared and analyzed with the accumulation time as the horizontal coordinate and the average force on seeds as the vertical coordinate at different times (Figure 3b). The average force of rice seeds with different moisture content showed a trend of increasing first and then decreasing during the accumulation process. At 10.23% of moisture content, the average force of rice seeds first rose to the maximum value. As the moisture content increased, the average force rose to the maximum value. The higher the moisture content, the later the average force rose to the maximum value. After the average force of rice seeds reached the maximum value, the average force of rice seeds with a moisture content of 10.23% firstly decreased and soon reduced to 0. With the increase of moisture content, the average force decreased to 0 in turn. The higher the moisture content, the time that the average force decreased to the lowest value shifted later. The changing trend of the average force of the seeds outside the cylinder at different times was consistent with the changing trend of the average velocity. This indicated that the force during the accumulation of the seeds was the direct cause of the change of their velocity.

**Figure 4:**
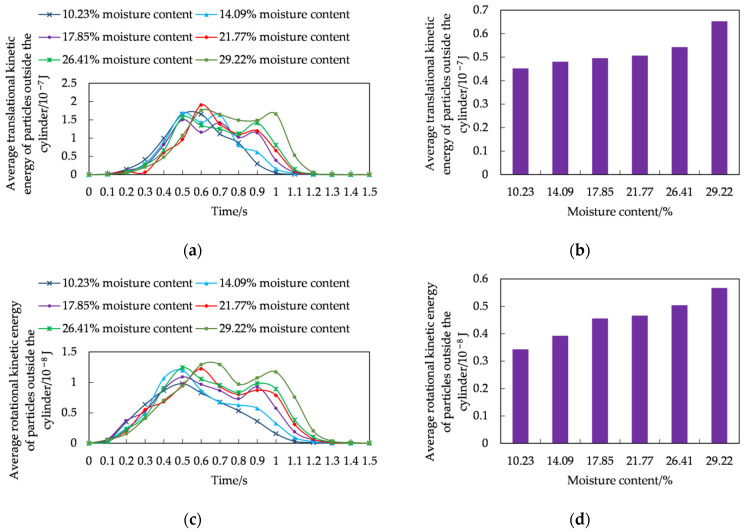
Average kinetic energy of rice seeds outside the cylinder: (**a**) average translational kinetic energy of rice seeds outside the cylinder at different times; (**b**) average translational kinetic energy of rice seeds outside the cylinder at the accumulation process; (**c**) average rotational kinetic energy of rice seeds outside the cylinder at different times; (**d**) average rotational kinetic energy of rice seeds outside the cylinder at the accumulation process.

To reveal the change of motion posture of the rice seeds with different moisture content during the accumulation process from the perspective of energy, the average kinetic energy of the rice seeds outside the cylinder was analyzed (Figure 4). As can be seen from Figure 4a,c, the overall translational kinetic energy was greater than the rotational kinetic energy during the accumulation of rice seeds, indicating that the sliding motion posture took up a larger proportion in the accumulation process. The average translational kinetic energy and average rotational kinetic energy of rice seeds with different moisture content showed a trend of first increasing and then decreasing, and the higher the moisture content, the longer the time of increasing and decreasing of translational kinetic energy and rotational kinetic energy.

The phenomenon was mainly due to the accumulation of rice seeds in the early stage of substrate support forming stage, and most of the rice seeds as a skeleton structure scattered in the bottom of the horizontal surface by comprehensive analysis of the speed and force during the accumulation of rice seeds. With the time increased, a large number of rice seeds formed the accumulation pattern and filled the spaces between the seeds, and the rice seeds became more prone to sliding or rolling. When there were no rice seeds down in the cylinder, no new rice seeds collided and pressed each other, the motion of the seeds was no longer active, and the average translational kinetic energy and average rotational kinetic energy gradually decreased after the free accumulation stage to the quasi-static accumulation stage. As can be seen from Figure 4b,d, the higher the moisture content, the higher the energy in the process of seed accumulation and the longer the time of kinetic energy decayed. This is explained from the side that the higher the moisture content, the longer the time of increase and decrease of the translational kinetic energy and rotational kinetic energy.

### 3.4. Analysis of Mechanical Characteristics of Rice Seeds in Quasi-Static Accumulation Stage

The rice seed system showed a certain static and stable state on the macroscopic level after the accumulation formation. However, the mechanical characteristics of the seed-to-seed and the influence law of the seed accumulation on the mechanical characteristics of the horizontal surface were still unclear. To analyze the mechanical characteristics of rice seeds in the quasi-static accumulation stage in detail, the seed accumulation was uniformly partitioned in a circular shape. The average value of the force on the seeds within a cylinder with a radius of 6 mm arranged in the center of the accumulation was the value of radial distance 0. The radial distance of 10 mm represented the average value of the force on the seeds within a circular cylinder with an inner diameter of 4 mm and an outer diameter of 16 mm, and so on. The partitions with radial distances of 0, 10, 20, ..., 140 mm were divided. The partitioning method is reported in Supplementary Materials (Figure S3a).

**Figure 5:**
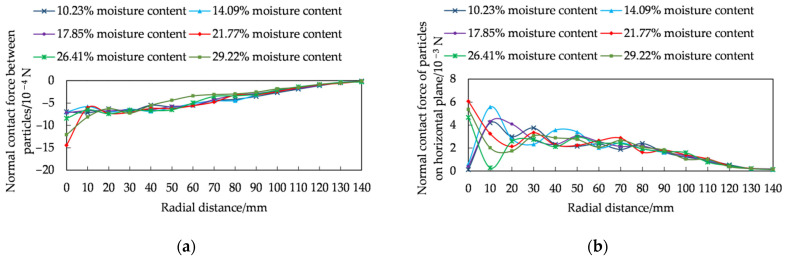
Analysis of mechanical characteristics of rice seeds in quasi-static accumulation stage: (**a**) normal contact force between rice seeds, (**b**) normal contact force on horizontal surface.

The variation of the normal contact force between the rice seeds along the radial distance was analysed (Figure 5a). The normal contact force between rice seeds with different moisture content was negative, indicating that the direction of contact force was downward. With the increase of radial distance, the normal contact force gradually decreased and reached 0 at the radial distance of 140 mm. This was mainly due to the fact that the amount of rice seeds in the area gradually decreased with the increase of radial distance, which led to the gradual decrease of the contact force between the seeds.

The contact force between the seed accumulation in the quasi-static accumulation stage was transferred to the bottom of the seeds through the force chain. The normal stress distribution at the bottom of the granular pile has always been a hot issue in the study of granular mechanics.

In this paper, the normal contact force on the horizontal surface of rice seed accumulation with different moisture content in radial position was extracted (Figure 5b). The normal contact force of rice seed accumulation with 10.23%, 14.09% and 17.85% moisture content to the horizontal surface was not the maximum value at the central part (radial distance of 0), but with the increased radial distance, the maximum value of normal contact force to the horizontal surface appeared at the position of the radial distance of 10 mm. The force distribution of the “central depression” structure was shown for rice seed accumulation with 10.23%, 14.09% and 17.85% moisture content. With the increased radial distance, the normal contact force on the horizontal surface gradually decreased, in which the second maximum peak of normal contact force occurred at the radial distance of 30 mm for 10.23% moisture content. The second peak of normal contact force occurred at the radial distance of 40 mm for 14.09% moisture content rice seeds. The 17.85% moisture content rice seeds at the radial distance of the second peak of normal contact force were observed at a radial distance of 50 mm. It showed that the peak of the second normal contact force became larger and larger from the center point as the moisture content increased. With the increased radial distance, the normal contact force on the horizontal surface gradually decreased and decreased to 0 at the radial distance of 140 mm. The normal contact force of the rice seed accumulation with 10.23%, 14.09% and 17.85% moisture content on the horizontal surface showed a maximum value at the center. Although the force value fluctuated with the increase of radial distance, the overall trend gradually decreased and reduced to 0 at the radial distance of 140 mm. The force distribution of the structure was no “central depression”.

The trend of normal contact force between rice grains was different from that of rice grains on the horizontal plane, which may be related to the fact that the shape of rice seeds and the form of interaction between seeds change the magnitude of the force and the form of force transfer. This changed the normal contact force acting on the horizontal plane, which was studied in depth by Zhao et al. [38].

**Figure 6:**
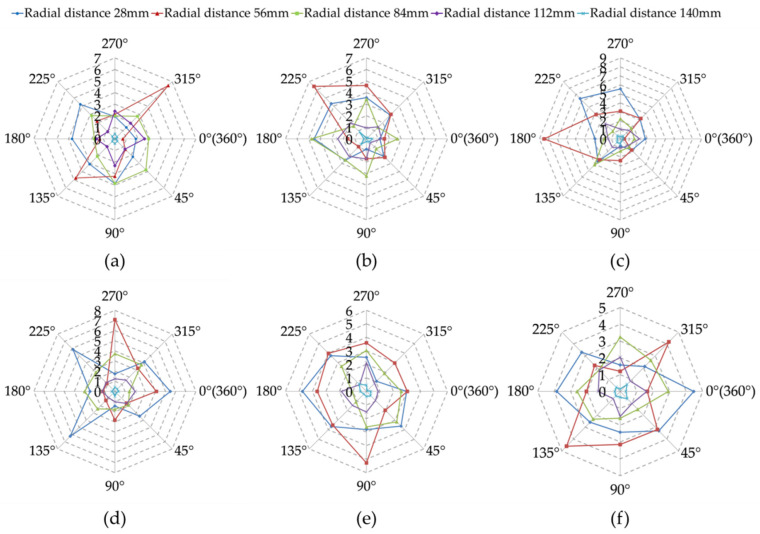
Normal force distribution on horizontal surface (unit: 10^−3^ N): (**a**–**f**) indicate the action of moisture content of 10.23%, 14.09%, 17.85%, 21.77%, 26.41%, 29.22% on the horizontal surface, respectively.

To show the distribution state of the contact force of the seed accumulation on the horizontal surface more clearly, the circular force at the bottom of the horizontal surface was divided into 8 discrete cells in a clockwise direction with the simulation *X*-axis as the horizontal zero point, and the center points along the radial direction were taken from the center of the circle of 14, 28, 42, 56, 70 mm on each discrete cell. The normal force of the bottom seed accumulation in the cell with the length of 14 mm and the width of 6 mm was taken at the center point. The normal force of the bottom seeds in the cell with a length of 14 mm and width of 6 mm was taken as the normal force of the horizontal plane with radial distance of 25, 56, 84, 112, 140 mm. The partitioning method is reported in Supplementary Materials (Figure S3b). The normal force distribution of the horizontal surface was analysed (Figure 6).

The distribution state of the force on the horizontal surface of rice seeds with different moisture content basically showed the same law. The distribution of the forces on the radial distance of 28 mm, 84 mm, 112 mm and 140 mm was relatively uniform, among which the force on the radial distance of 140 mm was the smallest. However, the force distribution fluctuated greatly at the radial distance of 56 mm, and there were extreme values. With the increased moisture content, the force at different radial distances gradually tended to a regular shape, indicating that the higher the moisture content of rice seeds, the more likely to appear the typical “ring force” structure, and the more uniformly the force on the horizontal surface was distributed in the circumferential direction. It was mainly due to the fact that the higher the moisture content of rice seeds, the larger the natural rest angle, the easier the seeds to move downward along the accumulation in the process of forming the accumulation, resulting in a certain “beat tight” effect along the surrounding area. Finally, a “ring force” structure with the cumulative effect of layers of seeds formed. When the moisture content was small, the natural rest angle formed by the accumulation of seeds was small, and the “beat tight” effect was not fully developed. Therefore, the “ring force” structure was not easy to appear.

Rice is one of the most important food crops in the world. The production, processing and storage of rice are of great significance to world economic stability and food safety [39]. This study focused on the physical characteristics of rice grains with different moisture content. The research results can be applied to the control and design of large grain unloading equipment and food processing receiving equipment. With the increase of moisture content, the time of substrate support forming stage and expansion accumulation stage becomes longer. That is, the rice grains with high moisture content cannot quickly enter the free accumulation stage, and the blockage problem is easy to occur. Therefore, when the unloading equipment is used to deal with rice with high moisture content, it is necessary to design a larger unloading port or raise a larger height to facilitate the discharge of rice grains. The greater the moisture content, the longer the kinetic energy attenuation time, the longer the time to reach the stable accumulation stage, and the longer the time required in the grain unloading stage. Therefore, in the automatic production line of food processing, when processing rice with large moisture content, the equipment needs to be set for a longer time to ensure the stability of grain unloading.

In addition, in the quasi-static accumulation stage, the pressure of low moisture content rice on the bottom has the characteristics of central depression. Therefore, it is necessary to reinforce the bottom plate at the non-central position at the bottom of the horizontal receiving tray of storage equipment or food processing equipment. The higher the moisture content, the greater the natural repose angle, the uniform stress in the circumferential direction of the bottom, and the smaller the surface area of the horizontal receiving tray. The rice grains with moisture content of 10.23%, 14.09% and 17.85% have the structure of central depression. For the food processing machinery with the accumulation center as the feed inlet, the blockage problem will occur. Therefore, the arc bottom groove and vibration are used to interfere with the stability of the central depression structure, so as to eliminate the arch effect of the indirect contact force of the particles and ensure the smooth entry of the grains into the feed inlet [12].

Rice seeds form the accumulation in a variety of ways, including “rainfall”, “dumping” and “point source” [17]. This paper studied the velocity and mechanical characteristics of rice seeds with different moisture content in the accumulation process and the mechanical characteristics of the quasi-static accumulation stage by taking the classical “point source” type as an example, showing that the accumulation characteristics of rice seeds with different moisture content were not the same, and not all rice seeds showed a significant “central depression” law in the normal force on the horizontal surface, which may be closely related to the physical characteristics of rice seeds. Rice with high moisture content needs drying treatment during long-term storage, otherwise, it is easy to lead to microbial development and mycotoxin formation. In the future, we will introduce different varieties of rice seeds as research objects to study the micromechanical characteristics of “long grain”, “medium grain” and “short grain”, and study the main factors influencing the mechanical distribution law of “central depression”, so as to further improve the system of research on the mechanical characteristics of rice seed accumulation. At the same time, the mechanism of microbial development and fungal formation during accumulation with different moisture content will be carried out to explore the changes of rice grains after accumulation from a chemical point of view. Combined with this study, a systematic and complete research system on the safe storage of rice grains will be formed from both physical and chemical aspects.

## 4. Conclusions

This study accurately established the model of rice seeds based on 3D scanning technology, simulated the accumulation state of rice seeds by “point source” accumulation method by discrete element method, and obtained the velocity, force, kinetic energy and force distribution under the accumulation state of rice seeds with different moisture content. The main conclusions are as follows:

(1) According to the velocity characteristics of rice seeds, the accumulation process can be divided into four stages: substrate support forming stage, expansion accumulation stage, free accumulation stage and quasi-static accumulation stage. The average velocity of rice seeds with different moisture content showed a trend of increasing and then decreasing during the accumulation process. The force during the accumulation of seeds was the direct cause of its velocity change.
(2) The average translational kinetic energy and the average rotational kinetic energy of rice seeds with different moisture content showed a trend of increasing and then decreasing, and the higher the moisture content, the greater the energy in the process of seed accumulation and the longer the kinetic energy decayed.
(3) The force distribution of the “central depression” structure of the action of rice seeds with the moisture content of 10.23%, 14.09% and 17.85% on the horizontal surface. The higher the moisture content of rice seeds, the more likely the typical “ring force” structure appeared, and the more evenly the force on the horizontal surface was distributed in the circumferential direction.
(4) When rice grains with 10.23% moisture content formed accumulation, the kinetic energy decay time was the shortest and the speed was the fastest; In the quasi-static accumulation stage, the force of rice grain with 21.77% water content on the horizontal plane is the most uniform, and it was the least prone to blockage when unloading at the accumulation center. This study provides a reference for the design and development of food processing equipment and safe storage equipment.

## Figures and Tables

**Table 1:** Mean values (±standard error) of measuring results of parameters at different moisture contents of rice seeds.

Parameters	10.23% Moisture Content	14.09% Moisture Content	17.85% Moisture Content	21.77% Moisture Content	26.41% Moisture Content	29.22% Moisture Content
Density/kg·m^−3^	910 ± 6.93 ^e^	966 ± 7.00 ^d^	990 ± 9.54 ^cd^	1005 ± 7.94 ^c^	1197 ± 4.00 ^b^	1253 ± 32.19 ^a^
Modulus of elasticity/MPa	252 ± 11.53 ^a^	220 ± 13.11 ^b^	211 ± 4.00 ^b^	208 ± 3.61 ^b^	170 ± 5.29 ^c^	102 ± 4.58 ^d^
Poisson’s ratio	0.38 ± 0.01 ^a^	0.37 ± 0.00 ^ab^	0.35 ± 0.01 ^bc^	0.33 ± 0.01 ^c^	0.30 ± 0.01 ^d^	0.25 ± 0.03 ^e^
Static friction coefficient *	0.28 ± 0.03 ^d^	0.30 ± 0.00 ^cd^	0.32 ± 0.02 ^bc^	0.32 ± 0.01 ^bc^	0.35 ± 0.01 ^b^	0.44 ± 0.03 ^a^
Dynamic friction coefficient *	0.015 ± 0.001 ^c^	0.017 ± 0.001 ^c^	0.026 ± 0.003 ^b^	0.026 ± 0.002 ^b^	0.028 ± 0.001 ^ab^	0.031 ± 0.001 ^a^
Collision recovery coefficient *	0.61 ± 0.02 ^a^	0.58 ± 0.01 ^ab^	0.56 ± 0.01 ^b^	0.43 ± 0.03 ^c^	0.38 ± 0.00 ^d^	0.30 ± 0.01 ^e^
Static friction coefficient **	0.44 ± 0.04 ^c^	0.48 ± 0.04 ^c^	0.51 ± 0.01 ^c^	0.56 ± 0.02 ^b^	0.60 ± 0.01 ^ab^	0.63 ±0.00 ^a^
Dynamic friction coefficient **	0.028 ± 0.002 ^d^	0.030 ± 0.001 ^d^	0.031 ± 0.000 ^d^	0.037 ± 0.002 ^c^	0.042 ± 0.001 ^b^	0.050 ± 0.003 ^a^
Collision recovery coefficient **	0.55 ± 0.03 ^a^	0.52 ± 0.02 ^a^	0.48 ± 0.01 ^b^	0.45 ± 0.00 ^b^	0.33 ± 0.02 ^c^	0.27 ± 0.03 ^d^

Different letters within the same row indicate a statistically significant difference (*p* < 0.05); * represents the measured value between rice grain and Q235 steel; ** represents the measured value between rice grain and rice grain.

## Data Availability

All data are presented in this article in the form of figures and tables.

## Supplementary Materials

The following supporting information can be downloaded at: https://www.mdpi.com/article/10.3390/foods11030295/s1, Table S1: Parameters measuring methods; Figure S1: The process of discrete element modeling of rice seeds; Figure S2: The results of bench test and simulation test of natural rest angle for rice seeds with different moisture content: (a1–a6) are the results of natural rest angle of bench test, respectively, and its moisture contents are 10.23%, 14.09%, 17.85%, 21.77%, 26.41%, and 29.22%, respectively. (b1–b6) are the result of the natural rest angle of EDEM simulation, and its moisture content are 10.23%, 14.09%, 17.85%, 21.77%, 26.41%, and 29.22%, respectively. The red dotted line is the primary fitting curve; Figure S3: Rice seed accumulation partitioning diagrams: (a) and (b) are the two partitioning methods respectively.
