# Supplementary material for: Discrete Element Simulation Study of the Accumulation Characteristics for Rice Seeds with Different Moisture Content
Source: Foods. 2022 Jan 22;11(3):295. doi: 10.3390/foods11030295 (PMC8834211; doi:10.3390/foods11030295)
Supplement: Supplementary file 1 [file foods-11-00295-s001.zip › foods-1518491-supplementary.pdf]

Table S1. Parameters measuring method.

Parameters	Measuring equipment	Measuring method	Calculating method
Moisture content	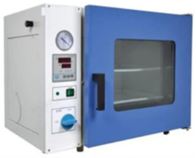 DZF-6051 electric blast drying oven (Qingdao Mingbo Environmental Protection Technology Co., Ltd., Qingdao, China)	The rice seeds were put into an aluminum box, weighed the pre-drying mass M_1, placed in the DZF-6051 type electric blast drying oven, placed continuously at 105°C for different times, removed the aluminum box, cooled to the room temperature, weighed the post-drying mass M_2, and calculated to obtain the moisture content C (%) of rice seeds.	$C = \frac{M_1 - M_2}{M_1} \times 100\%$
Density	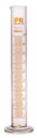 Measuring cylinder (Shanghai HuJiao Stationery Co., Ltd., Shanghai, China) 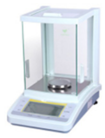 FA1004 electronic analytical balance (Shanghai HuJiao Stationery Co., Ltd., Shanghai, China)	Using FA1004 electronic analytical balance to select a quantitative amount of rice seeds respectively, their total mass M was determined; using the measuring cylinder to measure the volume of pure water V_1, the rice seeds were placed into the measuring cylinder to read the volume of the horizontal surface of the cylinder as V_2; the density of rice seeds can be calculated through the ratio of the total mass and volume difference.	$\rho = \frac{M}{V_2 - V_1}$

Modulus of elasticity	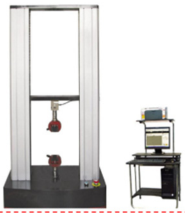<br>DX-500N electronic universal testing machine(Ningbo Dexun testing equipment Co., Ltd., Ningbo, China)	The rice seeds were placed on the rigid plate indenter of DX-500N electronic universal testing machine. the plate indenter was set to descend steadily at a constant speed. the rice seeds were compressed to the deformation. The load and displacement parameters at each moment were collected and recorded. The test was manually stopped loading when the load-displacement curve had a large abrupt change. the Hertz formula was used to solve the elastic modulus.	$E_V = \frac{0.338 K_V^{3/2} F(1-v^2)}{D^{3/2}} \left(\frac{1}{R_V} - \frac{1}{R_V^{'}}\right)^{1/2}$<br>where *F* is the loading load, N. *D* is the deformation of the rice seeds, mm. v is the Poisson's ratio of the rice seed. R_V and $R_V^{'}$ are the radius of curvature at the contact point on the upper surface of rice seeds during compression, mm. The rice seeds are subjected to the squeezing action of 2 rigid plate indenters in the thickness direction, and the principal radius of curvature at the contact point can be expressed as<br>$R_V = (W^2/4+T^2)/2T$,<br>$R_V^{'} = (L^2/4+T^2)/2T$. K_V is the coefficient determined by the radius of principal curvature. θ is the angle between the main plane at the point of contact between the upper surface of the rice grain and the rigid plate indenter, (°).
Static friction coefficient and dynamic friction coefficient	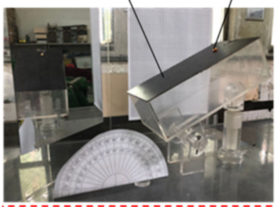<br>Test bench for measuring static friction coefficient	Static friction coefficient: The rice seeds were placed on the test plane and lift the test plane slowly until the rice seeds have a tendency to slide. The angle θ_1 between the formula plane and the horizontal plane was recorded. Dynamic friction coefficient: When the test material was tilted at a certain angle θ_2, the effective rolling displacement *L* and instantaneous velocity v_t of the movement of the	$\mu_1 = \tan\theta_1$<br>$\mu_2 = \tan\theta_2 - \frac{3v_t^2}{4gL\cos\theta_2}$

		center of the round corn roller along the inclined surface were measured by high-speed camera. The rolling friction coefficient between the rice seeds and each test material can be obtained according to the law of conservation of energy.	
Collision recovery coefficient	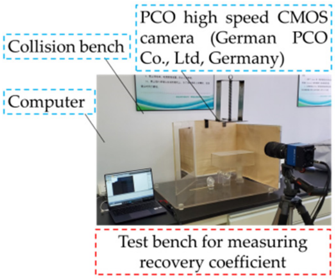	The collision platform was adjusted to the level, the rice seeds with a certain initial velocity rushed to the collision platform of the test material plate. The PCO high-speed CMOS camera on the spatial reference wall was used to the rice seeds spatial displacement measurement within reflective mirror. Collision recovery coefficient e can be obtained by converting the velocity ratio before and after the collision.	$e=\frac{v_t}{v_0}=\frac{2t_m\sqrt{v_x^2+v_y^2+v_z^2}}{2(z_m-z_0)+gt_m^2}$<br>where v_t is the instantaneous velocity of rice seeds rebounding after collision, m/s. v_0 is the instantaneous velocity of the falling rice seeds before the collision, m/s. v_x、v_y、v_z are the partial velocities of the rice seeds along *x*, *y* and *z* directions after collision, respectively, m/s. z_m and z_0 are the vertical coordinates before *m* frames and after the collision. t_m is the time from z_m to z_0, s.

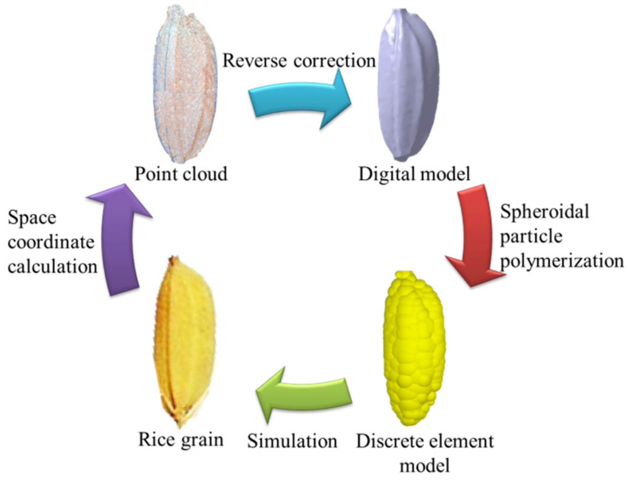

Figure S1. The process of discrete element modeling of rice seeds.

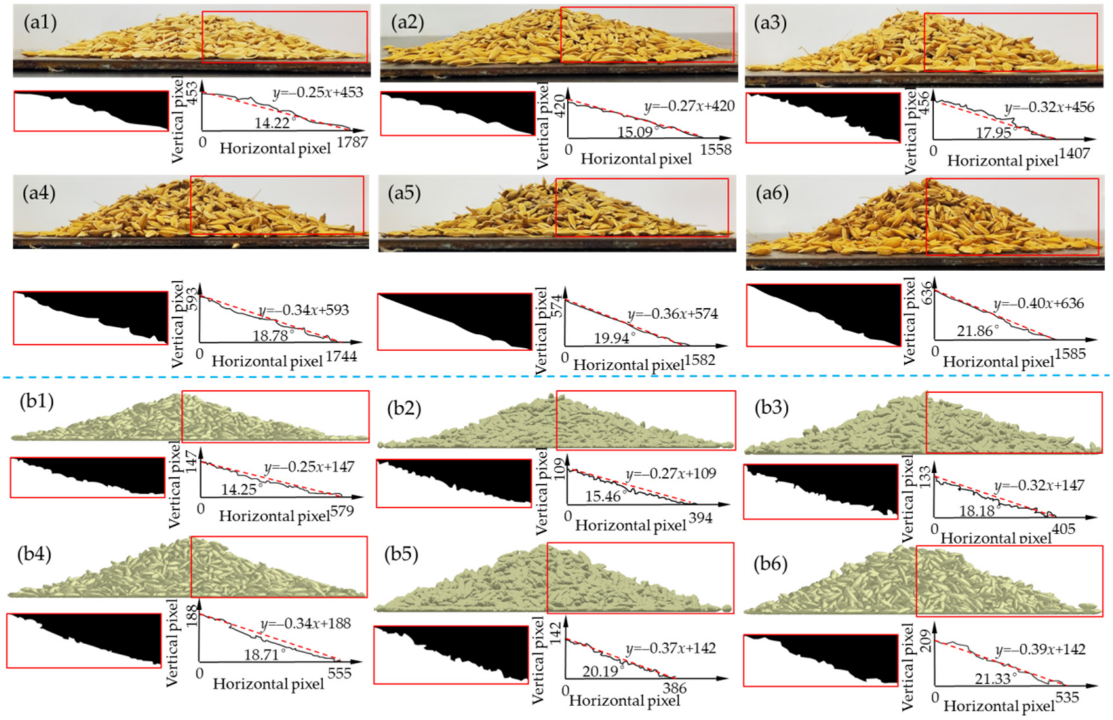

Figure S2. The results of bench test and simulation test of natural rest angle for rice seeds with dif-ferent moisture content are as follows: (a1)-(a6) are the result of natural rest angle of bench test, respectively, and its moisture content are 10.23 %, 14.09 %, 17.85 %, 21.77 %, 26.41 %, and 29.22 %, respectively. (b1)-(b6) are the result of the natural rest angle of EDEM simulation, and its moisture content are 10.23 %, 14.09 %, 17.85 %, 21.77 %, 26.41 %, and 29.22 % respectively. The red dotted line in the Figure 4 is the primary fitting curve.

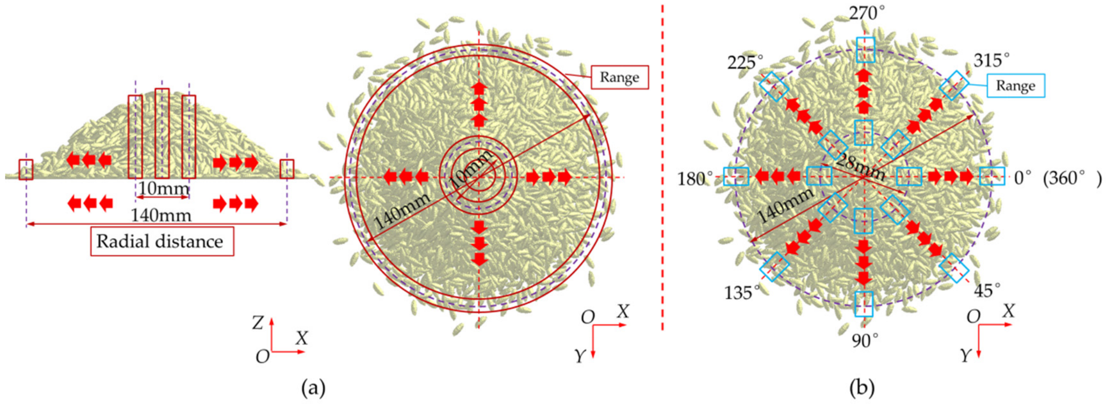

Figure S3. Rice seed accumulation partitioning diagrams: (a) and (b) are the two partitioning methods respectively.
